# Factors influencing repeated non-utilized healthcare appointments: a qualitative study of patient and provider perspectives in a Saudi outpatient setting

**DOI:** 10.1186/s12913-026-15260-0

**Published:** 2026-08-06

**Authors:** Asrar Aldadi, Kathryn A. Robb, Andrea E. Williamson

**Affiliations:** 1 School of Health and Wellbeing, University of Glasgow, Glasgow, UK; 2 College of Nursing, Taif University, Taif, Saudi Arabia

**Keywords:** Missingness, Qualitative research, Multiple no-show, Appointments utilization, Repeated non-attendance

## Abstract

**Introduction:**

Globally, multiple non-utilized appointments are linked to poorer health outcomes, increased emergency visits, higher hospitalization rates, gaps in preventive care, and increased mortality in patients with chronic conditions across primary and secondary healthcare. Studies of non-utilized appointments in Saudi Arabia have revealed barriers such as unequal access, lack of staff, lengthy wait times, and transportation issues leading to non-utilized appointments. However, no distinction has been made between single and multiple non-utilized appointments, or their causes in any depth. The aim of this study was to explore the factors contributing to multiple non-utilized appointments from the perspectives of both patients and healthcare providers, and to identify potential strategies for improving appointment utilization.

**Method:**

A qualitative study was carried out within different outpatient specialties in a hospital in Saudi Arabia. Twenty healthcare providers and twenty adult patients with multiple non-utilized appointments from clinics with high no-show rates were included. Semi-structured interviews were conducted in Arabic or English from December 2023 to April 2024. Data were analysed using inductive reflexive thematic analysis. The themes were aligned with Levesque’s access framework, which included both demand-side and supply-side perspectives.

**Results:**

Based on interviews with 40 participants (20 healthcare providers and 20 patients), the study identified five key themes contributing to multiple non-utilized appointments, with these factors affecting men and women differently: healthcare system determinants; patients’ attitudes and perceptions; communication support and engagement; health and well-being factors; and transportation, geographic and financial barriers. Potential interventions were grouped into two themes; firstly, enhancing healthcare delivery systems by improving appointment management and scheduling, implementation of a robust reminder system, improving communication system, optimizing staffing and improved collaboration with primary health centres. Secondly, improving patient accessibility and engagement; by enhancing transportation, fostering patient accountability, offering home visits, and enhancing patient motivation through educational initiatives and personal habit changes.

**Conclusion:**

This study showed a need for vital healthcare system changes both within hospital outpatient departments and in broader primary care services. Addressing non-utilized appointments requires a shift from isolated fixes to comprehensive, equity-driven approaches that consider both system efficiency and patients’ lived realities and abilities.

**Supplementary Information:**

The online version contains supplementary material available at https://doi.org/10.1186/s12913-026-15260-0.

## Introduction

Understanding the determinants and consequences of multiple non-utilized appointments is essential for improving healthcare delivery effectiveness and patient outcomes globally. The term “multiple non-utilized appointments,” defined as appointments made but not occurring two or more times as scheduled at the level of an individual patient, offers a more nuanced framework than stigmatizing terms like “no-shows” or “defaulters” that unfairly blame patients [1, 2]. This terminology shift recognizes the shared responsibility between patients and healthcare systems while acknowledging the complex factors contributing to appointment non-utilization.

The distinction between single and multiple non-utilized appointments is crucial for understanding the scope of this problem. Unlike single missed appointments which might stem from isolated incidents such as forgetting or unexpected emergencies [3], multiple non-utilized appointments suggest ongoing barriers to care access [4] and indicate deeper systemic engagement challenges. This pattern is consistent across healthcare contexts, patients who repeatedly miss general practitioner appointments also tend to miss outpatient appointments and experience “irregular discharge” from inpatient care, demonstrating a systemic engagement challenge [5].

The health consequences of this pattern are severe and far-reaching. Multiple non-utilized appointments are associated with poorer health outcomes [6–8], increased hospitalization rates [8], and premature mortality. Patients with long-term physical health conditions who miss appointments twice or more yearly see a threefold increase in all-cause mortality, while those with mental health conditions face an eightfold increase in risk of death [9]. Additionally, patients who consistently miss appointments raise concerns among clinicians as they are less likely to adhere to age-appropriate preventive health services [6], with 46% of frequent non-attenders having one or more chronic conditions [9].

Multiple non-utilized appointments are influenced by a complex interplay of demographic, social, personal, health-related, and system factors. Evidence on age is mixed [7, 10, 11], and although women attend GPs more often and miss multiple appointments, men miss a higher proportion overall [10]. Social determinants including frequent residential moves [11], disadvantaged areas [7, 11], and lower socioeconomic status [10, 12], consistently contribute repeated missed appointment. However, higher socioeconomic status may also increase missed appointments when patients use both private and public services [13]. Patient-related barriers include misconceptions about health conditions [14–18], low health knowledge [14, 18], and competing life demands [17–21]. System-level issues including long waits, poor clinic organization, lack of continuity, mistrust, referral miscommunication [16–18, 20–22], scheduling errors [23], short-notice appointments [20], insurance and interpreter use [6, 12], and structural booking constraints also play major roles [10]. These determinants reflect broader dimensions of healthcare access identified in the literature- Levesque’s framework-, encompassing the “approachability of services and patients’ ability to perceive their health needs; the acceptability of care and patients’ ability to seek it; the availability of services and ability to reach them; the affordability of care and ability to pay; and the appropriateness of services and patients’ ability to engage with them” [24].

Despite efforts to address missed appointments globally, it remains particularly pronounced in Saudi Arabian healthcare [2]. One recent development is that Saudi Arabia’s healthcare delivery system has increasingly incorporated digital solutions. In 2022, the Ministry of Health unified several platforms including Mawid and Sehha, into a single national app, Sehhaty, which has since reached over 27 million users and facilitated more than 11 million appointments [25]. The app enables patients to schedule appointments, access prescriptions, and consult providers remotely, and has been shown to reduce waiting times and improve care accessibility [26]. Virtual clinics and telemedicine are also well integrated, reducing the need for physical visits and increasing the likelihood of keeping scheduled appointments [27]. However, barriers including usability concerns, digital literacy gaps, and infrastructure limitations continue to affect engagement and drive missed appointments [27] (Table 1).

**Table 1:** Summary of digital health applications in Saudi Arabia

App name		Purpose	Function	User base
Sehhaty	Unified health platform providing healthcare services, health information, and integrated care	• Immediate consultations • **Booking and managing appointments** • Remote appointments • Search for medication • Digital Health Wallet • Vaccinations and health record tracking • Educational content • Prevention tools like weather warnings for asthmatics	Citizens, residents, dependents (children, elderly, people with special needs) in Saudi Arabia	
Mawid	Appointment booking and management feature within the Sehhaty app	• **Book**,** cancel**,** and reschedule appointments** • Centralized referral system • Tracks patient satisfaction and collects health data for planning	Primary healthcare patients	
Wasfaty	Medication dispensing and renewal app	• Links hospitals and pharmacies to ensure free and available medications • Enables patients to obtain medications without attending appointments	Patients in need of medication renewals	
Absher	Digital platform for Ministry of Interior services	• Integrated digital services for citizens, residents, and visitors. • Used for administrative and government services	Citizens, residents, and visitors	

In one 800-bed government hospital, non-utilization rates were as high as 57% in specialty clinics before quality improvement interventions, which still only reduced rates to 37%. Despite continuous quality improvement efforts in Saudi healthcare facilities, multiple non-utilized appointments persist as a significant challenge. There remains a notable gap in understanding this phenomenon within Gulf state countries’ unique cultural and healthcare contexts. No qualitative studies had examined the reasons behind multiple non-utilized appointments in Saudi Arabia [2].

This study investigated factors contributing to multiple non-utilized appointments in a Saudi hospital outpatient department and explored potential interventions to address them:

- What factors contribute to repeated non-utilized appointments in a general hospital in Saudi Arabia, and how do these factors differ between the perspectives of patients and healthcare providers?
- What interventions currently address multiple non-utilized appointments, how effective are they, and what recommendations do patients and healthcare providers suggest for improvement?

## Method

This qualitative descriptive study explored factors contributing to multiple non-utilized appointments and potential interventions from both patient and healthcare provider perspectives, guided by a social constructivist paradigm recognizing that individuals develop subjective meanings through social interactions and cultural contexts [28]. This design was chosen for its flexibility while providing an in-depth examination of the issue, allowing for a thorough analysis of multiple non-utilized appointments [29, 30].

The research was conducted in an 800-bed government hospital in Saudi Arabia with over 35 medical disciplines. Despite previous quality improvement initiatives reducing “no-show” rates multiple non-utilized appointments remained problematic.

As a Saudi female researcher (A. A.), I occupied both insider and outsider positions. My insider status as a Saudi citizen fluent in Arabic and English facilitated trust-building and cultural understanding. Being female was advantageous when interviewing women in a gender-segregated society. My tribal background (Banu Hudhayl) established immediate credibility and cultural connection with participants, and my younger age enabled younger participants to feel comfortable discussing sensitive topics. However, these characteristics also created potential barriers, as language differences with non-Saudi healthcare providers likely affected their responses and my interpretation of them. My insider status required conscious reflexivity to distinguish between participants’ actual perspectives and my own interpretations shaped by shared cultural background. Following phenomenological principles, I employed three strategies: bracketing assumptions (epoché), focusing on immediate experiences (description rule), and treating all data elements as initially equal (horizontalization rule) [31].

A heterogeneous purposive sampling approach was used to recruit participants [32–34]. Twenty healthcare providers were recruited from various outpatient clinics, positions, and countries, comprising nine women and eleven men. Twenty patients were recruited from four clinics with high non-utilization rates: Neurosurgery, Neurology, Psychiatry, and Diabetic Foot, with equal gender representation. One patient initially identified as a Psychiatry patient was found to have missed five appointments at the Cardiology clinic and was also included. A total of 103 potential patient participants were contacted, of whom 20 accepted and participated, six were excluded for not meeting inclusion criteria, 42 were non-contactable despite multiple attempts, and 35 declined. No participants withdrew after enrolment. The sample size of 40 participants was determined using information power principles, which prioritize data quality and variability over participant count [35, 36].

Ethical approval was obtained from the University of Glasgow Ethics and Governance Committee (Reference: 200230024) and the Saudi Arabian Ministry of Health in accordance with the Declaration of Helsinki. All participants received information sheets and privacy notices in both English and Arabic with informed consent obtained from all participants. Confidentiality was ensured through pseudonymization and removal of identifiers.

Semi-structured face-to-face interviews were conducted, typically lasting 30–60 min. Two topic guides were developed based on a systematic review of research on multiple non-utilized appointments [2], with separate versions for healthcare providers and patients (see Appendix 3). Both guides started with broad open-ended questions to encourage participants to share experiences freely. The healthcare provider guide explored perceptions of missed appointments, patient susceptibility patterns, current strategies, and intervention experiences. The patient guide examined attendance challenges, emotional responses, reasons for non-attendance, and suggestions for improvement. Each included follow-up prompts on communication barriers, transportation challenges, and competing priorities. Following the first two interviews Aldadi, Robb, and Williamson reviewed the interview transcripts for completeness and coverage of the study research questions. No revisions were required to the topic guide, so Aldadi proceeded with the rest of the interviews using the same questions. Interviews were recorded using an encrypted Dictaphone. Arabic was used for all patient interviews while healthcare provider interviews were conducted in either English [7] or Arabic [13]. Post-interview reflection forms and journals were used to systematically record the researcher’s observations, thoughts, and contextual notes immediately after each interview. Data collection occurred from December 2023 to April 2024.

Interviews were transcribed verbatim and Arabic interviews were personally translated by the researcher to preserve meaning and context [37]. Aldadi conducted the translations to maintain cultural context and meaning, and dialect congruence. A subset of four translations was reviewed by a bilingual academic colleague fluent in both languages to ensure accuracy and conceptual equivalence. The supervisors reviewed the first two translated interviews to also assess readability in English. All transcripts were pseudonymized and stored securely on the University’s One-Drive.

Reflexive thematic analysis followed Braun and Clarke’s six-phase approach was conducted [1, 38, 39] data familiarization through transcription and translation of all 40 interviews with translation applied to the 33 conducted in Arabic; [2] systematic inductive coding using NVivo; [3] grouping codes into initial themes; [4] developing and reviewing themes for consistency; [5] refining and defining themes in relation to research questions; and [6] report writing with participant quotations. Independent coding checks by supervisory team members covered 20% of transcripts. Findings were subsequently interpreted through Levesque’s healthcare access framework. Trustworthiness was ensured through: maintaining reflective journals, providing thick descriptions of the study context, regular supervision meetings, independent coding checks by supervisory team members (reviewing 20% of transcripts), and triangulation of patient and provider perspectives [28, 40].

## Results

### Participant characteristics

The study included 20 healthcare providers representing diverse specialties and nationalities across different clinical contexts (Appendix 1). Twenty adult patients who had experienced multiple non-utilized appointments were recruited from Neurosurgery, Neurology, Psychiatry, Diabetic Foot, and Cardiology clinics (Appendix 2). Patients aged 21–80 years with 2–11 missed appointments each, included Arabic-speaking Saudi and Yemeni participants (twelve married, four divorced, four single).

### Patients and healthcare providers perspectives on reasons for multiple non-utilized appointments

Five main themes grouped from the interviews, each with corresponding subthemes that illuminate the complex factors influencing appointment utilization (Table 2).

**Table 2:** Factors identified as influencing multiple non-utilized appointments

1.Patients’ attitudes, perceptions, abilities, and personal demands	2.Healthcare system determinants	3. Communication, support, and engagement in healthcare access	4.Health and well-being factors	5. Transportation, geographic, and financial barriers
**Sub-themes**	**Sub-themes**	**Sub-themes**	**Sub-themes**	**Sub-themes**
• Perceptions and opinions about terms	• Appointment management and procedures	• Language barriers and communication challenges	• Health conditions and medical factors	• Transportation challenges and accessibility
• Patients’ abilities and competing demands	• Resources management and services delivery challenges	• Family and gender dynamics	• Mental, emotional, and psychological factors	• Geographical barriers in healthcare access
• Misaligned perceptions, beliefs, awareness, and expectations about healthcare access	• Systemic barriers to continuity of care		• Stigma and privacy concerns	• Financial constraints and socioeconomic factors
^	• Technology and digital barriers	^	^	^

## Theme: patients’ attitudes, perceptions, abilities, and personal demands

### Perceptions and opinions about terms

The study revealed varied reactions to terminology. Some patients normalized terms like “no-show”, with one male patient stating, *“No*,* by God*,* on the contrary*,* I wouldn’t be upset if they referred me as the no-show patient. This means they’re interested and missed me” (Patient N6*,* male).* Similarly, several providers viewed these terms as factual descriptions: *“On the contrary*,* this increases society’s sense of responsibility. If you tell them ‘you missed your appointment’*,* they only came to renew it because they were absent in the first place*,* is that not right? So what is the problem with telling someone: ‘you did not attend your appointment’?” (Senior administrator N10*,* male).* However, many other patients experienced negative emotional reactions, feeling stigmatized or blamed, and some providers acknowledged that certain groups (women, elderly, educated patients) were more likely to react emotionally to these labels.

### Patients’ abilities and competing demands

Competing personal circumstances found as a primary reason for multiple non-utilized appointments, with most participants from both patient and provider groups identifying this as a key factor. As one male patient explained, *“The reasons that led to repeated absences are my circumstances and the circumstances of the family. Because I am the responsible person*,* sometimes I have work and I ask the hospital to book the appointment at a time that suits me and my work time*,* and they refuse” (Patient N6*,* male).* While some providers acknowledged these competing demands, others perceived patients’ absences as intentional, aimed at securing extended sick leave: *“The young ones who are absent*,* who are the government workers*,* break the fifth finger on the left hand. They all break their finger on purpose. Then he comes to take leave…” (Orthopaedic consultant N11*,* male).* This contrasted with patients’ perspectives, creating a mismatch in explanatory factors.

### Misaligned perceptions, beliefs, awareness, and expectations

Significant discrepancies appeared in how healthcare was perceived and valued. Many patients often lacked knowledge about hospital procedures, appointment importance, and health condition severity. Some believed traditional medicine was superior as one nurse observed: *“I mean*,* I had a patient who had a broken rib*,* so she told me to give her an X-ray on CD because she would take it to someone who would treat her with traditional medicine” (Nurse N17*,* female)*. Few healthcare providers reported patients perceiving the system as overly accommodating: *“Some patients are guaranteed to be served 1*,*000*,*000%. This is the fundamental point in every frequent patient absence. Some patients are not actually sick*,* yet they visit the doctor*,* who then clears them for discharge. However*,* they refuse to leave*,* insisting*,* “You do not have the right to discharge me. Give me another appointment”” (Nurse N16*,* female).* Additionally, some patients assumed appointments could be easily rescheduled, reducing their sense of commitment to attending, while some providers felt their professional role was reduced to supplying resources rather than delivering meaningful care, as patients sought alternative treatments elsewhere. A few participants from both groups perceived a gap that created a cycle in which patients felt dismissed, while providers attributed non-attendance to free healthcare reducing patient commitment.

## Theme: Healthcare system determinants

### Appointment management and procedures

Most participants from both groups identified scheduling challenges, including inconvenient appointment times, long delays, and inflexible policies, such as the closing of patient files after three missed appointments in a year (three-strike rule). While providers viewed this policy as necessary for effective scheduling, patients felt it was restrictive and added pressure to their appointment attendance. Calendar confusion was particularly problematic: *“Also*,* many people confuse the Hijri and Gregorian dates*,* as most Saudis primarily rely on the Hijri calendar.” (Nurse N17*,* female).* A significant contradiction was identified regarding appointment accessibility, several patients described difficulty securing appointments, while few providers believed easy access through apps and unofficial means contributed to non-attendance.

### Resources management and services delivery challenges

Facility limitations significantly impacted appointment utilization. Some patients struggled with crowded parking lots, sometimes waiting hours for a parking spot or parking far away, describing the long walk back as exhausting and resulting in lateness or missed appointments. Medication access problems also contributed to non-attendance, as reported by many participants from both groups: *“Sometimes. Because I don’t get any diabetes medication…Because I feel that there is no point in going to the appointment*,* as there is no medication at all” (Patient N2*,* female).* Several participants from both groups noted that overcrowded waiting areas and extended wait times further deterred attendance: *“From my perspective as a nurse*,* I often see patients getting upset and saying*,* “Why is the doctor taking so long?”” (Nurse N16*,* female)*. Additional barriers included inadequate support for disabled patients, delays in home medicine services, ineffective medications leading to repeated visits, and complex referral procedures with patients being transferred between multiple clinics. Some established care at primary centres and stopped attending hospital appointments, while procedural complexity in specialty clinics requiring multiple steps (blood tests, X-rays, weighing) before seeing doctors caused further frustration and non-attendance.

### Systemic barriers to continuity of care

Several participants from both groups identified the absence of efficient lines of communication and communication breakdowns between patients and providers significantly impacted care continuity. Some patients felt unheard, received unclear instructions, and faced dismissive attitudes when asking questions. Administrative errors were common: *“You put a certain file in*,* and it becomes a mess. Tomorrow*,* they might put people in the operating room who have no connection to the issue*,* while the main patient who should be there is ignored because the wrong file was allocated” (Patient N13*,* female).* Improper attendance recording leading to false “no-show” records, frequent physician changes requiring patients to repeatedly explain their medical history, and misdiagnoses causing loss of trust were additional challenges. Furthermore, the doctor-nurse hierarchy created tension, whereby patients tolerated unprofessional behaviour from doctors yet dismissed nurses’ guidance and instructions, undermining nurses’ authority and leading to multiple non-utilized appointments. Some participants indicated that these systemic failures resulted in patients seeking care elsewhere, using personal connections to bypass the system, or abandoning treatment altogether.

### Technology and digital barriers

Digital health applications presented both opportunities and challenges. Few healthcare providers noted that medication access through apps reduced attendance: *“Honestly*,* the “Wasfaty” system is messing up all the appointments. Now*,* people don’t even have to attend in person; they just get their prescriptions through the “Wasfaty” app and end up missing their appointments” (Psychiatric consultant N9*,* female).* Additional barriers included inconsistent reminder systems with delayed or incorrect notifications, frequent SIM card replacements causing missed alerts, and confusion from conflicting information between the Sehhaty app and text messages. Many patients received no instructions on app usage, while some hospital specialties remained unlinked to the Mawid system, preventing reminders entirely, contributing to no-show rates reaching 30% in some clinics.

## Theme: Communication, support, and engagement in healthcare access

### Language barriers and communication challenges

Language differences created significant obstacles, particularly for international patients, as reported by some providers and some patients. As one consultant explained: *“We could not communicate with this patient because he speaks a specific Bangladeshi language. We even tried to communicate with him through the nurse*,* through the cleaners*,* but we could not” (Internal medicine consultant N13*,* male).* Some patients relying on non-medical staff like cleaners for translation, family members serving as interpreters during appointments, and overseas staff unfamiliar with Arabic or cultural needs. Critical miscommunications occurred when incorrect contact numbers were registered, leading to reports of missed essential follow-up tests. Saudi staff often incorrectly assumed all patients, including children, could communicate in English, creating further barriers to effective care delivery and appointment attendance.

### Family and gender dynamics

Family support played a crucial role in appointment attendance. For few women, family control sometimes presented significant barriers: *“Sometimes there are problems between me and my husband. When I ask him to take me to my appointment*,* he refuses and says*,* ‘By God*,* you will not get out of here’. If I get psychological pressure*,* I can’t move*,* I get very tired and I can’t go” (Patient N2*,* female).* Several participants from each group identified additional challenges included patients with disabilities or mobility issues lacking assistance, elderly patients depending on family members who did not understand appointment importance, and women having to secretly attend appointments to avoid confrontation. Despite a few providers observing a gradual shift toward women’s independence in healthcare management, family members remained essential as translators, advocates, and for transportation assistance, particularly for patients with language barriers or those from less educated backgrounds.

## Theme: Health and well-being factors

### Health conditions and medical factors

Physical health directly impacted appointment utilization. Chronic conditions and fatigue were commonly cited barriers by many patients: *“By God*,* the most common thing is*,* as I told you*,* I am sick and I am tired*,* and in fact this thing makes me tired at one point*,* so I cannot walk much. My Lord is All-Knowing*,* Glory be to Him. Yes*,* my legs are tired*,* my body is tired*,* sometimes I am like you say*,* the pressure rises on me” (Patient N2*,* female).* Dialysis patients being too exhausted to attend same-day appointments, patients weighing 30–35 kg feeling too frail to travel, and unexpected hospitalizations disrupting scheduled visits were additional factors. Many delayed seeking care until conditions became severe, fearing they would burden the system. Conversely, some patients with intermittent issues like back pain viewed appointments as optional when feeling better, missing crucial follow-ups that could prevent complications like improper bone healing or nerve damage. This pattern particularly affected elderly patients who did not find their conditions bothersome enough to warrant regular monitoring.

### Mental, emotional, and psychological factors

Both groups experienced emotional responses to missed appointments. Few providers expressed frustration: *“I feel upset because it wastes employees’ time. I waited to assist the patient*,* but they never showed up” (Rehabilitation medicine doctor N7*,* male).* Fear and anxiety about medical procedures deterred many patients, particularly for endoscopy appointments. Depression was a significant barrier, especially for many women attending urology and psychiatric clinics. Additional factors included few patients feeling guilty yet relieved when missing appointments, fear of serious diagnoses like cancer, anxiety about multiple X-rays and blood tests, and COVID-19 pandemic concerns. Some patients showed indifference, viewing appointments as non-critical, while many others experienced frustration from frequent treatment modifications and inadequate equipment. Negative staff interactions and fear of penalties created feelings of helplessness and resentment, ultimately discouraging future attendance.

### Stigma and privacy concerns

Mental health stigma significantly affected appointment attendance for many patients. One nurse described a patient traveling long distances to avoid being seen at local facilities: *“That patient is working in another city*,* and his family is also there. Yet he travels specifically to our hospital for his appointments*,* doing everything he can to attend. But why does he not choose a hospital in his own city? because of the stigma” (Nurse N15*,* female).* Several providers similarly noted that stigma often prevented patients from attending necessary appointments, as they feared being seen at mental health clinics and judged by their families and communities. So, a few patients scheduled appointments in early morning to avoid family suspicion, kept mental health conditions secret for extended periods (up to a year), for fear of community gossip and judgment.

## Theme: Transportation, geographic, and financial barriers

### Transportation challenges and accessibility

Mobility issues significantly affected appointment attendance, especially among most elderly patients and those with disabilities: *“Yes*,* the first thing is the wheelchair. This is one of the difficulties. I mean*,* there is no getting in the car*,* getting off*,* or getting in” (Patient N1*,* female).* There were additional barriers such as limited public transportation with distant meeting points, unavailable ride-hailing services in suburban/rural areas, vehicle breakdowns, and scheduling conflicts. Several women faced disproportionate challenges due to limited independent transport options. While some providers believed transportation was not a major issue in Saudi Arabia since many women now drive, significant disparities were reported as remaining for elderly patients, those with disabilities, and residents of remote areas who struggled with unreliable transport services and lack of wheelchair-accessible vehicles.

### Geographical barriers in healthcare access

Distance between residence and healthcare facilities presented significant challenges for several patients. As one patient stated: *“The hospital’s location is far away. The hospital is in the city and I am in the village” (Patient N6*,* male).* Additional barriers included travel times of 45 min to an hour, with some patients travelling from remote areas hundreds of kilometres away, and the burden of multiple trips for various healthcare needs including pharmacy visits. Weather conditions like rain further impeded access sometimes.

### Financial constraints and socioeconomic factors

Economic limitations prevented some patients from attending appointments. One female patient explained: *“Yes*,* I can’t take a taxi every now and then for 40*,* 50*,* or 60 riyals. Sometimes I swear it costs 120 riyals to get me to the hospital and another hospital. After I pay money*,* I don’t get anything or any benefit from the doctor. I don’t have a job. This is difficult” (Patient N13*,* female).* Few providers acknowledged these challenges: *“Yes*,* I think so. Some people in remote areas struggle due to their financial situation. They cannot afford to book transportation or may not have the means to travel here” (Nurse N3*,* female).*

### Theoretical implications related to factors influencing multiple non- utilized appointments

Levesque’s five accessibility dimensions of, ‘approachability’ in this study affected by fear, language barriers, and complex digital systems, ‘acceptability’ influenced by perceived judgment and lack of empathy from providers, ‘availability and accommodation’ limited by rigid appointment systems and geographical barriers, and ‘appropriateness’ undermined by negative provider interactions and unmet needs, directly impacted patients’ abilities to effectively access care helps frame this study in the wider literature and our analysis of solutions.

From the patients’ perspective, ‘ability to perceive’ was compromised by competing priorities, perceived ease of rescheduling, limited health literacy, and misinterpreting symptom improvement as recovery. ‘Ability to seek’ was hindered by limited women’s autonomy, inadequate family support, social stigma, provider distrust, depression, and anxiety. ‘Ability to reach’ was affected by family dependence for navigation of care, psychological barriers from chronic conditions, rural transportation challenges, and frequent SIM changes causing missed notifications. Despite free healthcare, the ‘ability to pay’ was constrained by indirect costs like transportation and lost wages, disproportionately affecting lower-income patients. The ‘ability to engage’ was undermined by mental health struggles, negative responses to medical terminology, traditional medicine preferences, and distressing waiting conditions. This analysis revealed how multiple non-utilized appointments result from complex interactions between systemic inefficiencies and patient constraints within Saudi Arabia’s cultural context (Table 3).

**Table 3:** Summary of findings mapped to Levesque’s framework

Levesque’s Dimension	System-Level Factors (Approachability, Acceptability, Availability, Appropriateness)	Patient-Level Factors (Ability to Perceive, Seek, Reach, Pay, Engage)
Approachability / Ability to perceive	• Fear • Language barriers • Complex digital systems	• Competing priorities • Perceived ease of rescheduling • Limited health literacy • Misinterpreting symptom improvement as recovery
Acceptability / Ability to seek	• Perceived judgment • Lack of empathy from providers	• Limited women’s autonomy • Inadequate family support • Social stigma • Provider distrust • Depression • Anxiety
Availability / Ability to reach	• Rigid appointment systems • Geographical barriers	• Family dependence for navigation of care • Psychological barriers • Rural transportation challenges • Frequent SIM changes causing missed notifications
Affordability / Ability to pay	^	• Indirect costs such as transportation • Lost wages • Disproportionately affecting lower-income patients
Appropriateness / Ability to engage	• Negative provider interactions • Unmet needs	• Mental health struggles • Negative responses to medical terminology • Traditional medicine preferences • Distressing waiting conditions

### Interventions and recommendations to address appointment non-utilization

Two main themes were identified regarding interventions and recommendations to improve appointment utilization:

## Theme: Enhancing healthcare delivery systems

### Improved appointment management and scheduling

Current interventions included a policy of closing patient files after three missed appointments and 24-hour rescheduling windows. Some patients requested better flexibility: *“They should allow scheduling at least five times so the patient can make their appointment. Or schedule the same method*,* but five times*,* not just once*,* because we can’t predict life’s circumstances” (Patient N16*,* female).* Additional improvements included morning and evening clinic options, WhatsApp lines for missed appointments. However, communication gaps meant several patients were unaware of this service. Further accommodations included special arrangements for couples to coordinate their appointments simultaneously, reducing the burden of multiple trips, and elderly patients, and providers booking follow-up appointments during consultations. Tensions arose between consolidating multiple appointments for efficiency versus scheduling separate visits to avoid rushing patients. These accommodations often depended on individual provider initiatives rather than standardized policies, highlighting gaps between hospital procedures and patient needs for accessible, timely care.

### Implementation of a robust appointment reminder system

Various reminder methods were used including paper reminders with calendar information, phone calls for high-priority patients, and text messages. Several patients and a few healthcare providers recommended consistent automated reminders: *“I received a message at night. I mean*,* they are good in just saying*,* “You did not attend.” Ok*,* just send the appointment to us*,* send the appointment*,* give us a call. I mean*,* if they make a call*,* it won’t hurt them and say*,* “You have an appointment like this and this.”.” (Patient N19*,* female).* Additional practices included providers using personal phones for reminders, specialty-specific approaches (neurology receiving consistent calls while ophthalmology only for surgery), and dual calendar systems causing confusion. Few healthcare providers with international experience described different reminder approaches from their previous work settings. A provider who had worked in India explained they relied on manual systems using hospital cards and patient self-tracking rather than electronic reminders. Similarly, a provider with Greek experience noted that phone call reminders were standard practice in private clinics but not used in public healthcare facilities, highlighting how reminder system implementation varies across different healthcare contexts.

### Effective communication system

Some patients and providers emphasized the importance of maintaining accurate personal data to prevents appointment errors, and enhance communication and service delivery. One patient stated; *“My first advice is to ensure that mobile numbers are accurate and patient names are correct. There should be no confusion between departments*,* for example*,* a patient should not be scheduled in one clinic while they are actually supposed to be in another.” (Patient N13*,* female).* Frequent SIM card changes remained a persistent challenge requiring multiple layers of verification to ensure consistent patient communication. In response, additional measures were needed such as mandatory contact updates (though these were inconsistently implemented across the system) and point-of-service verification systems where patients update their contact information via forms during emergency department visits. Few providers emphasized personalized communication for elderly patients and caregivers while translation services were available through hospital staff serving dual roles.

### Optimizing staffing and training for enhanced patient care

Several participants from both groups recommended increasing healthcare professional provision in clinics and enhancing training in communication skills, psychological support, and patient-centred care. As one female ophthalmologist explained: *“The problem is we need a [nursing] sister to assist us. We would be very fast in the clinic*,* because we are the ones doing everything*,* calling the patient*,* examining the patient*,* writing in the system*,* and giving appointment” (Ophthalmology specialist N18*,* female).* Key issues included single doctors serving multiple-room clinics, patient preferences for culturally-sensitive local providers, and lack of awareness that the Patient Affairs Department could help patients switch to different healthcare providers. Some patients also prefer attentive doctors who provided psychological support and thorough explanations particularly in cardiology and psychiatry. Providers recognized nurses’ critical role beyond direct care including system management and appointment scheduling, while few patients prioritized punctuality and organization from their healthcare providers.

### Improving efficiency and accuracy in patient management

Few providers reported efforts to manage patient flow: *“They reduce the number of patients for the clinic. Our clinic has 20 patients. Ok*,* the patient needs to talk to the doctor*,* at least he can see the doctor for 6 minutes” (Nurse N16*,* female).* Key strategies included discharging non-urgent cases, targeted capacity adjustments in specialty clinics, and overbooking systems that added 10 extra appointments to compensate for no-shows. International comparison revealed a contrasting approach, as one provider observed: “We need to have only 10–15 patients so we can focus more on the patients. In Angola, there is too much clinic, too much doctor. So it is good” (Ophthalmology specialist N18, female). Priority card systems for elderly and disabled patients faced implementation challenges with misuse creating longer waits and disabled parking access issues for some patients. Several patients highlighted critical gaps including inconsistent diagnostic protocols between consultants, incomplete medical records hindering emergency care, and insufficient consideration for elderly patients despite priority status. Clinics that require multiple procedures such as internal medicine, dental care, and ophthalmology struggled with adequate time allocation per patient.

### Enhancing outpatient department management

Equipment reliability directly impacted appointment utilization for some patients. One patient stated: *“We need more heart monitors—there is only one available*,* and this is the difficulty I’m facing. The pressure gauge is broken*,* which is annoying” (Patient N17*,* female).* Other key challenges included electronic check-in machines creating confusion and erroneously marking present patients as absent, while the ticket system inadvertently penalized punctual patients. The disconnect between paper appointment times and electronic queue numbers frustrated some patients who arrived on time but were seen after later arrivals who checked in earlier. Extended clinic hours (8 AM-4 PM) eliminated midday breaks but patients still requested evening options. Privacy concerns particularly affected psychiatric clinic attendance for some patients due to open layouts visible to passers-by. Malfunctioning equipment sometimes forced postponements directly contributing to non-utilized appointments. Reliance on intermediaries (e.g., cousins, tribal members, hospital contacts) for managing appointments reduced patient accountability, as many patients felt others would reschedule for them. To address this, some providers recommended limiting intermediaries to improve attendance.

### Optimizing hospital and health centre collaboration

Few participants from both groups suggested improving primary care utilization: *“They must activate the role of the health centre. They must make two shifts*,* morning and evening*,* in which the health centres provide treatments for people. The crowding at the hospital will decrease” (Patient N6*,* male).* Key recommendations included increasing hospital capacity through private hospital partnerships, implementing payroll-deducted health insurance for private care access, and establishing specialized neighbourhood centres like a successful diabetes care model. Providers advocated for a formal “referral back” system to return stable patients to primary care without criticism. Medication management was identified as a critical factor with patients requesting more efficient distribution and clearer instructions for long-term conditions. Some providers suggested linking medication dispensing to appointment attendance to improve compliance, mirroring psychiatric clinic practices.

## Theme: Improving patient accessibility and engagement

### Enhancing transportation and accessibility for patients

Several patients and a few providers suggested dedicated transportation services. Key initiatives included the “Kayan” app providing free transportation coupons (though the contract had expired and was awaiting renewal), proposals for hospital-funded transport particularly for elderly patients, and shuttle/train services from key locations. Parking challenges were reported as significant barriers, with suggestions for multi-level parking on the hospital’s ground-level-only campus. Beyond physical infrastructure, societal barriers particularly affected women’s healthcare access, with family restrictions preventing attendance. One patient noted: *“Look*,* you know the root of the problem is my family*,* right? I mean*,* if I gave up on them*,* I’d expect that I wouldn’t even need to go to a clinic—I’d be completely cured. If I lived away from my family and stayed at home alone*,* I’d be fine with going to appointments.” (Patient N16*,* female).* Furthermore, mobility difficulties experienced by some unsteady patients highlighted the need for companion services.

### Advancing virtual appointments for enhanced accessibility

The hospital implemented virtual consultations though opinions on their effectiveness varied. One senior nurse highlighted their efficiency: *“Virtual clinics are very convenient*,* if used correctly. The patient does not need to bother with me by arriving*,* pulling a number*,* and waiting at the clinic for a period of time” (Senior nurse N4*,* male).* Success varied by specialty with endocrinology clinics thriving while psychiatry found limitations for severe conditions. The Sehhaty app facilitated follow-ups without physical visits particularly benefiting elderly patients by reducing travel and wait times. However, resistance was expressed from several older patients preferring traditional examinations with stethoscopes and tests, viewing remote consultations as inadequate “jokes”. Digital literacy gaps and trust issues prevented some from utilizing these services. While suitable for stable conditions and general advice, virtual appointments faced scepticism regarding their validity without in-person diagnostic procedures.

### Establish patient accountability, expectations and responsibilities

Some providers emphasized being firm about attendance: *“It is better for us employees in the appointments department to be really strict and punctual. You see*,* if you do not come to your appointment*,* I will not renew it for you*,* even if he is my biological brother” (Senior administrator N10*,* male).* They suggested linking consequences to missed appointments to encourage attendance. Providers personally applied strict policies by refusing to renew appointments for repeat no-shows, directing them through standard re-referral processes. A penalty system similar to driver’s license violations was proposed to incentivize attendance. Cultural factors complicated enforcement as free healthcare services created hesitance to “force” compliance. Few providers noted the importance of patients respecting allocated appointment days while acknowledging the delicate balance between firmness and cultural sensitivity.

### Offer home visits when appropriate

Some patients emphasized the need for more accessible home-based care: *“Home medicine. Every time I am tired*,* they prepare for me. Medical staff or home medicine means that whenever I call them*,* they come and see me and they help me a little. However*,* my family is tired*,* and every time I get tired*,* I get shortness of breath. I mean*,* I could die before I reach the hospital” (Patient N1*,* female).* Despite existing services, significant gaps remained in addressing patient needs. Current quarterly visits were perceived as insufficient for those requiring more frequent care, particularly during acute episodes. Few patients highlighted life-threatening risks of hospital travel for chronic conditions, advocating for on-demand services without scheduled constraints. Cardiology and diabetes clinics were suggested as referral points for systematic home medicine enrolment, and those with transportation difficulties, mobility limitations, or family burden. The program’s restrictions frustrated patients who needed proactive physical assessments but were too unwell for hospital visits.

### Enhancing patient motivation

Some providers suggested incentives for punctual patients and positive communication: *“The white lie means*,* as they say*,* the white lie is necessary*,* meaning we say*,* “No*,* your matters are good*,* God willing*,* there is improvement”. On the contrary*,* this encourages me to continue treatment*,* and to continue*,* and attend my appointments” (Administrator N12*,* male).* Motivational strategies included complimentary gifts for consistent attendance and compassionate care approaches. These providers emphasized how optimistic communication, even in severe cases, maintained hope and treatment adherence. Personal experiences with family members reinforced the value of encouraging words in sustaining patient commitment to scheduled appointments.

### Promote appointment attendance through personal habits and practical strategies

Few patients described strategies to improve attendance, including learning from others with similar conditions: *“If someone reassures me more*,* it would help. Also*,* if I learn from the experiences of people who have gone through the same struggles as me and confirm that we share the same condition*,* I would definitely value their opinions” (Patient N20*,* male).* Personal strategies included maintaining healthy routines like early sleep schedules, achieving financial stability, and spacing appointments to avoid daily overload. Patients valued peer support and shared experiences as motivators. Some linked appointments to significant dates like religious occasions as memory aids.

### Deliver educational initiatives

Few participants from both groups emphasized the importance of health education. One patient suggested: *“I swear*,* an idea came to my mind. I mean*,* did you see that when we enter the hospital*,* we find them doing*,* for example*,* education about asking God for safety*,* a certain disease*,* breast cancer*,* how can one avoid it? How*,* when you do education*,* for example*,* about absence*,* what will it do? Educate patients” (Patient N7*,* female).* Providers used social media platforms (X, Snapchat) for outreach through posters and videos explaining booking procedures. Educational needs varied: Bedouin communities required targeted awareness programs, families needed condition-specific management training (anxiety, Parkinson’s), and patients wanted app navigation guidance. Suggestions included periodic multimedia campaigns with sound-enabled screens, face-to-face sessions for questions, and clear signage. Some patients remained passive, relying on faith or routines, highlighting awareness gaps about appointment importance.

### Theoretical implications related to interventions for non-utilized appointments

Levesque’s healthcare access framework also offered valuable theoretical insights for understanding interventions aimed to reduce non-utilized appointments in the Saudi healthcare context. On the system side, interventions to improve ‘approachability’ included multi-channel communication strategies and informal translation support through bilingual staff; ‘acceptability’ was enhanced through dual-calendar reminders (Hijri and Gregorian) and culturally competent care training; ‘availability and accommodation’ was addressed via streamlined protocols and integrated appointment systems; and ‘appropriateness’ was improved through standardized scheduling guidelines and specialty-specific virtual appointment protocols.

Correspondingly, interventions targeting patients’ abilities included educational demonstrations about virtual appointments and culturally sensitive health initiatives ‘ability to perceive’; flexible booking systems and mobility services for women ‘ability to seek’; partnerships with “Kayan” services and evening clinics to reduce wage loss ‘ability to pay’; and digital literacy training for older adults and peer support groups to enhance motivation ‘ability to engage’. This comprehensive theoretical analysis demonstrating the usefulness of Levesque’s framework highlights how effective interventions must address both healthcare system accessibility dimensions and patients’ corresponding abilities (Table 4).

**Table 4:** Interventions to address multiple non-utilized appointments mapped to Levesque’s framework

Levesque’s Dimension	System-Level Interventions	Patient-Level Interventions
Approachability / Ability to perceive	• Multi-channel communication strategies • Informal translation support through bilingual staff	• Educational demonstrations about virtual appointments • Culturally sensitive health initiatives
Acceptability / Ability to seek	• Dual-calendar reminders (Hijri and Gregorian) • Culturally competent care training	• Flexible booking systems • Mobility services for women
Availability / Ability to reach	• Streamlined protocols • Integrated appointment systems	^
Affordability / Ability to pay	^	• Partnerships with Kayan services • Evening clinics to reduce wage loss
Appropriateness / Ability to engage	• Standardized scheduling guidelines • Specialty-specific virtual appointment protocols	• Digital literacy training for older adults • Peer support groups to enhance motivation

## Discussion

### Summary

This qualitative study exploring multiple non-utilized appointments in a Saudi general hospital revealed five key contributing factors: patients’ attitudes, perceptions, abilities and personal demands (appointment rescheduling assumptions, forgetfulness, work commitments); healthcare system determinants (scheduling inefficiencies, overcrowded clinics, technological barriers); communication support and engagement in healthcare access (language barriers, family involvement); health and well-being factors (physical exhaustion, intentional non-attendance for perceived non-urgent conditions, stigma); and transportation, geographic and financial barriers (lack of personal and public transportation, extensive travel distances, socioeconomic factors). These findings broadly align with established Saudi healthcare literature, where studies consistently identified lack of knowledge about health conditions, lower education levels, forgetfulness, work commitments, and household responsibilities as barriers to healthcare access across various outpatient setting [41–47]. Similarly, our results parallel global research identifying patient-related factors, healthcare system determinants, and communication challenges as key contributors to multiple non-utilized appointments [2]. These findings demonstrate that multiple non-utilized appointments stem from complex interactions between systemic challenges and patients circumstances rather than simply patient choices to not attend.

The study also examined interventions addressing multiple non-utilized appointments, identifying two main themes: enhancing healthcare delivery systems (reminder systems, scheduling policies, staff training) and improving patient accessibility and engagement (transportation services, educational initiatives, digital literacy support). While other evidence across Saudi healthcare settings demonstrates the effectiveness of such interventions for instance, a cross-sectional study of 211 patients at internal medicine outpatient clinics in Saudi Arabia recommended enhanced appointment reminder systems as a key strategy to address forgetfulness [42], and a tertiary hospital in Saudi Arabia demonstrated that automated reminders sent 24 and 48 h before appointments significantly reduced no-shows and waiting times [48], their efficacy in this study varied due to inconsistent implementation across departments, communication gaps between providers and patients, and accessibility barriers for certain demographics. Saudi-specific implementation barriers were also identified, including dual calendar system complications, three-strike policies conflicting with international patient-centred approaches, service discontinuity issues with transportation apps, and elderly patient resistance to virtual appointments. These findings highlight the need for comprehensive, culturally appropriate interventions that address both healthcare system limitations and patients’ ability to navigate them effectively.

The Levesque healthcare access framework provided valuable theoretical insights by revealing how multiple non-utilized appointments result from complex interactions between healthcare system deficiencies and patient-related challenges. For instance, hospital communication systems ‘supply-side’ often failed to adapt to patients’ frequent SIM card replacements ‘demand-side’, while rigid policies like the three-strike rule prioritized system efficiency over patients’ unpredictable circumstances. Previous Saudi research has similarly documented that scheduling systems frequently failed to provide suitable appointment times, contributing to prolonged wait times and increased congestion in outpatient departments [41, 47]. The framework also highlighted cultural mismatches, such as confusion between Hijri and Gregorian calendars and resistance to technological solutions among older patients, demonstrating how supply-side interventions can fail without accounting for patients’ cultural expectations, abilities, and health beliefs.

The framework further illuminated differential impacts across patient groups. Beyond general gender disparities documented in the Saudi literature where research has highlighted gaps in addressing women’s mental health needs and restrictions on healthcare autonomy shaped by societal norms [49, 50], our study uncovered complex transitional dynamics where traditional dependency patterns coexist with emerging autonomy. Women reported needing to “sneak out” for healthcare while providers acknowledged increasing independence among newly licensed female drivers, creating tension between cultural expectations and evolving social norms. Elderly patients and those with lower educational levels similarly struggled with navigating digital systems and understanding appointment instructions, a challenge reflected in Saudi telemedicine research showing that older individuals, women, and lower-income groups demonstrated lower adoption of digital health platforms [51]. These insights underscore the importance of developing interventions that address both systemic inefficiencies and patient constraints while considering cultural context and the specific needs of vulnerable populations.

### The digital paradox in healthcare access

Our study uncovered a critical digital paradox in Saudi healthcare access where technological systems (Sehhaty, Mawid, Wasfaty) designed to enhance accessibility paradoxically created new barriers. While Saudi literature documents general digital health adoption issues [51–54], our study identified specific technological barriers including Hijri-Gregorian calendar confusion causing appointment misunderstandings, contradictory information between Sehhaty app and SMS notifications, and the paradoxical use of Wasfaty app in psychiatric settings where medication refills were intentionally restricted to ensure continued patient visits. These findings revealed that implementation without adequate user support and cultural adaptation actually impaired access rather than improving it. The ease of digital rescheduling we observed paradoxically reduced patients’ sense of medical urgency, transforming appointments from critical healthcare encounters into optional administrative tasks. This technology-access paradox demonstrates that digital solutions in Saudi healthcare require user-centred design maintaining human connection alongside digital convenience with interventions co-designed by both providers and patients to bridge the disconnect between automated systems and patients’ need for personalized approaches.

### Contribution to the field

This study advanced understanding of multiple non-utilized appointments in healthcare by clarifying terminology confusion and showing through Levesque’s framework that multiple non-utilized appointments indicate deeper access issues. It uncovered unique Saudi cultural factors affecting attendance, including male guardianship impacts on women’s access and Hijri/Gregorian calendar confusion. Creative strategies were documented, such as providers using personal phones for reminders.

### Implications for research and practice

This study highlights that healthcare access in Saudi Arabia is shaped by complex personal, cultural, and systemic factors. Multiple non-utilized appointments are not just administrative issues, they reflect deeper challenges in both healthcare delivery and patient experience. A central cross-cutting finding was the stark perception gap between providers and patients regarding appointment non-utilization. These gaps appeared across multiple domains: patients emphasized their diligent attendance efforts while providers perceived intentional absences for sick leave; patients felt appointment systems were inflexible while providers believed easy access through apps contributed to multiple non-utilized appointments; and patients valued the “Wasfaty” app’s convenience while providers saw it as contribute to multiple non-utilized appointments. This perception gap helps explain why many isolated interventions fail solutions designed from one perspective may not address the actual barriers experienced by the other group.

Effective solutions must be systemic and patient-centred. On the supply side, improvements include integrated booking systems, better communication strategies, culturally appropriate reminders, and tailored virtual care protocols. In relation to communication barriers, our findings suggest the need for formalized professional medical interpretation services rather than reliance on informal translators. On the demand side, interventions should focus on health education, digital literacy training, and support services such as transportation and family involvement. Therefore, addressing multiple non-utilized appointments requires a shift from isolated fixes to comprehensive, equity-driven approaches that consider both system efficiency and patients’ lived realities.

### Strengths and limitations

Up to our knowledge this study represents the first in-depth examination specifically focused on multiple non-utilized appointments in Saudi outpatient departments, using comprehensive methodology with reflexive thematic analysis. The diverse sample of 20 patients from five clinics and 20 providers provided rich comparative perspectives. The researcher’s Saudi background and bilingual skills facilitated trust while maintaining objectivity. The study captured both formal interventions and creative informal practices developed by participants.

However, the small sample of 40 participants from a single hospital limits generalizability. The study had minimal representation of non-Arabic speaking patients, while language barriers with non-Saudi providers affected data quality. The researcher’s background may have influenced participant responses. As a cross-sectional study, it captured only a snapshot of evolving interventions without quantitative effectiveness metrics. Finally, the theoretical framework did not fully address certain issues like provider burnout and resource distribution that impact healthcare access. Levesque’s framework could be further developed to include a dimension addressing systemic infrastructure and resource allocation to encompass factors like provider burnout and equipment functionality which were identified as barriers.

## Conclusion

This thesis explored multiple non-utilized appointments in Saudi Arabia’s healthcare system, finding they result from complex systemic and personal factors rather than patient irresponsibility. Through patient and provider interviews it clarifies terminology and uncovers cultural factors like calendar confusion and gender dynamics affecting healthcare access. The study shows how digital tools can create barriers despite good implementation.

Using Levesque’s framework revealed interactions between patient abilities and system factors. Effective solutions must address both system inefficiencies and patient barriers through dual-calendar reminders, flexible scheduling, tailored support for vulnerable groups, and improved communication. Technology adoption must consider digital literacy to avoid worsening access disparities.

This research provides insights for administrators and policymakers, and is of particular relevance for those supporting Gulf healthcare understanding and Saudi Vision 2030 goals. Future research should test interventions, study patients with multiple long term conditions, examine gender barriers, address mental health stigma in culturally sensitive ways, and explore how healthcare providers manage complex systems.

## Supplementary Information

Below is the link to the electronic supplementary material.

Supplementary Material 1

## Data Availability

The datasets generated and analyzed during the current study are not publicly available due to participant confidentiality and privacy concerns but are available from the corresponding author on reasonable request.
