# Supplementary material for: Factors influencing repeated non-utilized healthcare appointments: a qualitative study of patient and provider perspectives in a Saudi outpatient setting
Source: BMC Health Serv Res. 2026 Aug 6;26:1525. doi: 10.1186/s12913-026-15260-0 (PMC13732855; doi:10.1186/s12913-026-15260-0)
Supplement: Supplementary file 1 [file 12913_2026_15260_MOESM1_ESM.docx]

Appendix 1: Healthcare providers’ demographic characteristics

| Participant ID | Gender | Nationality | Specialty | Current position at the hospital | Years of experience at this hospital | Location of Education | Previous work Experience |
| --- | --- | --- | --- | --- | --- | --- | --- |
| N.1 | Male | Middle East | Medical social work | Director of Patient Affairs-appointments manger | 6 Years | Saudi Arabia | 16 Years in Saudi |
| N.2 | Male | Middle East | Medical social work | Director of Patient Experience | 7 Years | Saudi Arabia | 13 Years in Saudi |
| N.3 | Female | South- eastern Asia | Nurse | Quality coordinator | 12 Years | Philippines Country | 2 Year in Philippines- 3 years in Saudi Arabia |
| N.4 | Male | Middle East | Nurse | OPD supervisor | 17 Years | Saudi Arabia | 4 Years in Saudi Arabia |
| N.5 | Male | Middle East | Occupational medicine doctor | Occupational medicine specialist | 2 Years | Saudi Arabia | 12 Years in Saudi |
| N.6 | Female | South Asia | Nurse | Specialist nurse | 7 Years | India | 2.5 Years in India |
| N.7 | Male | North-east Africa | Rehabilitation medicine doctor | Physician | 10 Years | Egypt | 23 Years in Egypt |
| N.8 | Male | Middle East | General practitioner | Director of OPD | 7 Years | Saudi Arabia | 2 Years in Saudi |
| N.9 | Female | North-east Africa | Psychiatry | Psychiatry consultant | 4 Years | Egypt | 12 Years in Saudi- 9 Years in Egypt |
| N.10 | Male | Middle East | Medical records technician | Medical record supervisor | 10 Years | Saudi Arabia | 3 Years in Saudi |
| N.11 | Male | North Africa | Orthopaedic doctor | Consultant | 7 Years | Tunisia-Paris | 12 Years in Saudi |
| N.12 | Male | Middle East | Health administration | Appointment coordinator in OPD | 3 Years | Saudi Arabia | 12 Years in Saudi |
| N.13 | Male | North-east Africa | Internal medicine doctor | Consultant | 2 Years | Sudan | 6 Years in Saudi |
| N.14 | Male | Southern Europe | Orthopaedic doctor | Consultant | 2 Years | Greece and UK | 28 Years in Greece and UK |
| N.15 | Female | South Asia | Nurse | Quality coordinator | 9 Years | India | 3 years in India |
| N.16 | Female | Middle East | Nurse | Nurse | 11 Years | Saudi Arabia | None |
| N.17 | Female | North-east Africa | Nurse | Nurse | 2 Years | Sudan | 11 Years in Sudan |
| N.18 | Female | North America | Ophthalmology doctor | Consultant | 7 Years | Cuba | 2 Years in Anguilla- 11 Years in Cuba |
| N.19 | Female | Middle East | Dental assistant | Dental assistant | 5 Years | Saudi Arabia | 3 Years in Saudi |
| N.20 | Female | Middle East | Nurse | Nurse | 11 Years | Saudi Arabia | 1 Year |

Appendix 2: Patients’ demographic characteristics

| Participant ID | Age | Gender | Nationality | Marital status | Number of no-shows | Clinic |
| --- | --- | --- | --- | --- | --- | --- |
| N.1 | 76 | Female | Saudi | Married | 2 | Diabetic foot |
| N.2 | 50 | Female | Saudi | Married | 5 | Diabetic foot |
| N.3 | 74 | Male | Saudi | Married | 11 | Diabetic foot |
| N.4 | 80 | Male | Saudi | Married | 3 | Diabetic foot |
| N.5 | 63 | Female | Saudi | Married | 6 | Diabetic foot |
| N.6 | 59 | Male | Saudi | Married | 3 | Neurology |
| N.7 | 50 | Female | Saudi | Married | 2 | Neurology |
| N.8 | 35 | Female | Saudi | Divorced | 2 | Psychiatry |
| N.9 | 29 | Male | Yemeni | Married | 2 | Neurology |
| N.10 | 56 | Male | Saudi | Married | 2 | Neurology |
| N.11 | 37 | Female | Yemeni | Divorced | 2 | Neurology |
| N.12 | 22 | Male | Saudi | Single | 2 | Psychiatry |
| N.13 | 43 | Female | Saudi | Single | 5 | Cardiology |
| N.14 | 33 | Male | Saudi | Married | 2 | Neurosurgery |
| N.15 | 30 | Male | Saudi | Single | 3 | Psychiatry |
| N.16 | 21 | Female | Saudi | Single | 2 | Psychiatry |
| N.17 | 60 | Female | Saudi | Divorced | 5 | Neurosurgery |
| N.18 | 38 | Male | Saudi | Married | 2 | Neurosurgery |
| N.19 | 65 | Female | Saudi | Divorced | 2 | Neurosurgery |
| N.20 | 40 | Male | Saudi | Married | 2 | Neurosurgery |

Appendix 3: Interview questions

**Initial interview questions**

I. **Healthcare provider interview questions:**
1. In your opinion, what are the main reasons for patients repeatedly missing their appointments ('No-shows')?

- Do you think practical issues like competing priorities e.g. word, family commitments play a role in patients missing appointments?
- What extent do you believe hospital communication contributes to patients missing their appointments repeatedly? Specifically, how do various aspects of hospital communication, such as appointment reminders, scheduling procedures, and information dissemination, impact patients' attendance rates and the likelihood of missed appointments?
- Do you think transportation is a factor in patients missing appointments repeatedly?
- Are there circumstances where patients might prioritize other things over their appointments?
- Can you share any specific instances or stories that highlight these reasons?

2. Do you think there are any particular patient groups that might be more susceptible to repeated “missed appointments” due to these reasons?
3. What specific strategies or measures does your department currently employ to effectively handle challenges such as practical issues, communication barriers, and competing priorities, if any?
4. Have you encountered any interventions in your entire work experience that were implemented to reduce missed appointments?
5. What interventions have been implemented at this hospital to reduce missed appointments?
6. What do you believe is the most effective way to improve and excel?
7. What is it like for you when your patients missed their appointments repeatedly?

- How does that make you feel?
- What do you do when your patients missed their appointments repeatedly?
- What do you think the impact of a missed appointment is on the patient?
- Do patients have any awareness of their appointments?

8. How do you perceive the terms "missed appointments" and "no-show"? Do you think these terms carry any negative connotations or stigma for patients who experience them?
9. Have you noticed any reactions or emotional responses from patients when they are referred to as having "missed appointments" or labeled as a "no-show"? How do you interpret these reactions?
10. Is there anything you would like to add to regarding issues linked with “No-show”?
II. **Patients interview questions:**
1. Attending hospital appointments can be challenging for some people, do you have any experience of finding it difficult to attend a hospital appointment?
2. How do you feel when you miss an appointment?
3. Thinking back, what are the reasons for missing these appointments repeatedly?

- Some people think that communication challenges, competing priorities, health-related issues, practical reasons, personal commitments, financial constraints and transportation challenges can contribute to multiple missed appointments? Do any of these reasons resonate with your experience, or were there other factors that played a role in your case?

4. What do you do next when you have missed appointments?
5. Are repeatedly missing appointments connected to anything else in your life?
6. What sort of things do you think might help you to attend appointments in the future?
7. What could the hospital do to support you in attending appointments??
8. Do you think that being labeled as having "missed appointments" or "no-show" in the past has influenced your decision to continue attending or missing appointments?
9. Have you noticed any change in your feelings or motivations toward attending appointments after being referred to as a "missed appointment" or "no-show"? If yes, could you elaborate on how these terms have affected you?
10. Is there anything you would like to add?
